# Co-detection in the pathogenesis of severe hand-foot-mouth disease

**DOI:** 10.1007/s00705-012-1396-6

**Published:** 2012-07-13

**Authors:** Li-Juan Liu, Hong-Mei Xu, Xiu-Jun Li, Jun Wang, Xian-Jun Wang, Shu-Jun Ding, Fang Tang, Jing Wang, Ying-Jie Zhang

**Affiliations:** 1 Institute of Health Quarantine, Chinese Academy of Inspection and Quarantine, Beijing, 100123 People’s Republic of China; 2 Department of Diarrhea, Children’s Hospital, Chongqing Medical University, Chongqing, 400014 People’s Republic of China; 3 Department of Epidemiology and Health Statistics, Shandong University, Jinan, 250012 People’s Republic of China; 4 Department of Infectious Disease Control, Shandong Provincial Disease Prevention and Control Center, Jinan, 250001 People’s Republic of China; 5 Center for Diseases Control and Prevention of Chinese Peoples’ Armed Police Forces, Beijing, 102613 People’s Republic of China; 6 National Center for Public Health Surveillance and Information Services, Chinese Center for Disease Control and Prevention, 155 Changbai Road, Changping District, Beijing, 102206 People’s Republic of China

**Keywords:** Hand-foot-mouth disease, enterovirus 71, co-detection

## Abstract

It still needs to be elucidated whether co-detection of EV71 with other intestinal tract viruses plays a role in the pathogenesis of severe hand-foot-mouth disease (HFMD). A total of 680 stool specimens collected from clinically diagnosed mild and severe-HFMD patients were tested for EV71, CA16, norovirus, bocavirus and rotavirus. The results showed that EV71 was significantly associated with severe-HFMD patients. Co-detection of EV71 with norovirus and rotavirus was also significantly associated with severe-HFMD patients: The OR (95 % CI) value was 6.466 (2.735, 15.283) and 7.561 (3.560, 16.057), *p* < 0.001, respectively. Co-detection of EV71 with rotavirus or norovirus is probably associated with severe HFMD.

Hand-foot-mouth disease (HFMD) is a cluster of manifestations with fever and exanthema of the hands, feet and palm, which is mild, and recovery occurs 4-6 days in most instances [1–3]. It is spread by fecal-oral transmission, and it prevails all over the world. Previous studies have demonstrated that enterovirus 71 (EV71) and coxsackievirus A16 (CA16) were the common agents of the disease; however, after EV71 infection, only a small proportion of the patients developed severe HFMD, accompanied by neurological complications, cardiopulmonary collapse or death. In recent decades, HFMD has become severe public-health concern, especially in the Western Pacific Region [1, 4, 5]^.^ The largest and most severe EV71-associated HFMD outbreak took place in Taiwan in 1998, when 405 pediatric HFMD patients developed severe neurological complications and pulmonary edema, and 78 children died [6]. In mainland China, several outbreaks of HFMD with severe complications have been reported since 2003, and the numbers of reported cases are increasing. Even in the first half of 2010, approximately one million cases were reported, including 15,501 severe cases and 537 deaths [7].

Clinical observations revealed that severe HFMD occurred in only a small percentage of HFMD patients, and EV71 was identified as the major agent [8–11]. Since the virus was primarily isolated from stool specimens and the intestinal tract, which are was complicated surroundings containing multiple pathogens, we suspected that besides EV71, other intestinal tract viruses exist that may be involved in the process of severe HFMD in susceptible infants and young children. Therefore, in the present study, we detected EV71 as well as bocavirus (BV), rotavirus (RV) and norovirus (NV) in patients with mild and severe HFMD to investigate the potential role of these viruses in the pathogenesis of severe HFMD.

During the period from May to December 2010, clinically diagnosed HFMD patients from two children’s hospitals were selected as the study subjects. The two hospitals, located in the cities of Chongqing and Jinan are the largest pediatric hospitals in Sichuan and Shandong Province, respectively. All of the patients with HFMD who were admitted to the hospital were treated by professional pediatricians. Detailed medical histories and results from clinical examinations and laboratory tests were extracted from medical records to confirm the hospital diagnosis. Pediatric HFMD was confirmed when a child had oral ulcers and vesicular rash on the hands, feet, knees, or buttocks. None of the patients had typical symptoms of watery diarrhea. Severe-HFMD patients were defined as those with neurological complications or cardiopulmonary symptoms that developed after the initiation of HFMD without other causes. Neurological complications included lethargy, a tendency to panic, headache, vomiting, limb myoclonus, nystagmus, ataxia, eye movement disorders, weakness and acute flaccid paralysis. Stool specimens were collected from all of the patients and stored at -20 °C until detection. Informed consent was obtained from the guardians of all of the patients, and the study was reviewed and approved by the ethics committee of the Chinese Academy of Inspection and Quarantine.

Total viral RNA was extracted from stool specimens using the a QIAamp MinElute Virus Spin Kit (QIAGEN, Hilden, Germany). Detection of EV71 and CA16 were performed using commercial kits for EV71 and CA16 (KingHawk Co. Ltd, China). Some of the EV71-positive amplicons were sequenced and analyzed by phylogenetic analysis using MEGA (version 3.1). The statistical significance of the inferred phylogenies was estimated using bootstrap analysis with 1000 pseudo-replicated datasets. The nucleotide sequences generated in the study were deposited in GenBank under accession nos. HQ668351- HQ668343, HQ668341, HQ668333, HQ668307, HQ668304 and HQ668296. Bocavirus and norovirus assays were performed based on Taqman real-time PCR and real-time RT PCR systems, with the primers and probes designed as described elsewhere [12, 13]. Rotavirus was detected using an IDEIA rotavirus A group direct antigen detection kit (IDEIA, Oxiod, UK) following the instructions of the manufacturers.

Pearson’s chi-square test and one-way analysis of variance (ANOVA) were used for analyzing categorical data and continuous data, respectively. Multivariate logistic regression analysis was used to detect the relationship between severe-HFMD patients and detected viruses separately, after being adjusted for age and gender. Relative excess risk of interaction (RERI) and attributable proportion due to interaction (AP%) were used to estimate the join effect of co-detection of EV71 with other viruses in the severe-HFMD patients [14].

$$ {\text{RERI}} = {\text{RR}}\left( {\text{AB}} \right) - {\text{RR }}({\text{A}}\overline{\text{B}} ) - {\text{RR }}(\overline{\text{A}} {\text{B}}) \, + 1;{\text{ AP}}\,\% = \frac{{[RR(AB) - RR({\text{A}}\overline{\text{B}} \, ) - {\text{RR}}( \, \overline{\text{A}} {\text{B}}) + 1]}}{{{\text{RR}}({\text{AB}})}} $$

A *P*-value of less than 0.05 was considered statistically significant. All of the analysis was performed using the SPSS 18.0 software package.

During the study period, a total of 680 patients with HFMD were recruited as the study subjects from two provincial children’s hospitals, including 237 girls and 443 boys, with a male-to-female ratio of 1.9:1, and the mean age of the study patients was 29 months (range: 7 to 51 months).

Of the patients, 244 were diagnosed as having severe HFMD, including 230 patients with neurological complications and 14 with other complications. The mean age of the severe-HFMD patients was 28 months, which was insignificantly lower than 30 months for the mild-HFMD patients (*p* > 0.05). Of the patients with severe HFMD, 70 % were male, whereas only 63  % of the mild cases were in males. However, no significant difference was observed between the two groups (*p* > 0.05).

Univariate analysis showed that single infection with EV71, norovirus or rotavirus was detected with significantly higher frequency in severe-HFMD patients, in comparison with mild-HFMD patients (*p* < 0.001). After adjusting for age and gender, the association between severe-HFMD and EV71, norovirus, and rotavirus remained the same. In contrast, CA16 was significantly underrepresented in the severe-HFMD patients (*p* < 0.001) (Table 1).

**Table 1:** Logistic analysis of the relationship between the detected viruses and severe HFMD

	No. (%) of HFMD cases	No. (%) of severe HFMD cases	OR (95 % CI)^a^	P^a^	OR (95 % CI)^b^	P^b^
EV71	152 (34.9 %)	134 (54.9 %)	1.900 (1.317,2.740)	0.001	2.372 (1.716,3.278)	0.000
CA16	148 (33.9 %)	51 (20.9 %)	0.698 (0.457,1.066)	0.096	0.499 (0.345,0.721)	0.000
BV	18 (4.1 %)	15 (6.1 %)	1.434 (0.694,2.962)	0.330	1.444 (0.712,2.930)	0.309
NV	31 (7.1 %)	31 (12.7 %)	2.074 (1.203,3.575)	0.009	1.885 (1.112,3.195)	0.019
RV	31 (7.1 %)	42 (17.2 %)	2.633 (1.587,4.368)	0.000	2.823 (1.714,4.649)	0.000

a, unadjusted by age and gender; b, adjusted by age and gender, CI, confidence interval

The rate of co-detection of EV71 with other viruses in severe-HFMD patients was estimated separately for norovirus, rotavirus and bocavirus, using the same strategy. The patients were subdivided into four groups according to the presence or absence of these viruses, and the relative risk of severe-HFMD patients was estimated with the non-infection group as a reference in the multivariate logistic analysis model, with age and gender being adjusted. No significant association was seen for the EV71-BV co-detection group, and the OR value and 95  % CI were estimated as 0.868 (0.339, 2.225). A significant association was seen for the EV71-NV co-detection group and the EV71-RV co-detection group, and the OR and 95 % CI were estimated as 6.466(2.735, 15.283) and 7.561(3.560, 16.057), respectively, *P* < 0.001. The RERI and AP% were calculated as 3.765, 58 % and 4.365, 58 %, respectively, for the EV71- NV and EV71- RV co-detection groups (Table 2).

**Table 2:** EV71 co-infection with other viruses in severe HFMD cases

		No. (%) of HFMD cases	No. (%) of severe HFMD cases	RR (95 % CI)^a^	P^a^	RERI	AP%
EV71	BV						
-	-	276 (63.3 %)	105 (43 %)	1.000			
-	+	8 (1.8 %)	9 (3.7 %)	0.374 (0.147,0.950)	0.039		
+	-	143 (32.8 %)	120 (49.2 %)	1.112 (0.298,4.149)	0.875		
+	+	9 (2.1 %)	10 (4.1 %)	0.868 (0.339,2.225)	0.769		
EV71	NV						
-	-	264 (60.7 %)	98 (40.2 %)	1.000			
-	+	23 (5.3 %)	12 (4.9 %)	1.390 (0.663,2.914)	0.383		
+	-	140 (32.2 %)	115 (47.1 %)	2.311 (1.642,3.254)	0.000		
+	+	8 (1.8 %)	19 (7.8 %)	6.466 (2.735,15.283)	0.000	3.765	58 %
EV71	RV						
-	-	274 (60.9 %)	84 (36.5 %)	1.000			
-	_+_	21 (4.7 %)	15 (6.5 %)	2.011 (0.992,4.076)	0.053		
+	-	144 (32 %)	104 (45.2 %)	2.185 (1.544,3.093)	0.000		
+	+	11 (2.4 %)	27 (11.7 %)	7.561 (3.560,16.057)	0.000	4.365	58 %

a, adjusted by age and gender; CI, confidence interval. RERI, relative excess risk index; AP%, attributable proportion

We further evaluated the joint effects of the co-detection status using the algorithms of both the additive and multiplicative models. Under the additive model, we expect the joint excess rate of the two viruses will be equal to the sum of the excess rate from each virus separately. Under the multiplicative model, we expect the joint rate ratio of the two viruses to be equal to the product of the rate ratios for each virus separately. The close agreement for the observed joint rate and that expected under the multiplicative model suggests that the relationship between viral co-detection and severe HFMD is closer to being multiplicative than to being additive.

Our present data, as reported in previous studies, showed that EV71 was significantly associated with severe HFMD. In addition, we present evidence for the first time that co-detection of EV71 with other intestinal-tract viruses could pose a substantial risk of severe HFMD. Particularly for the simultaneous detection of EV71-NV and EV71-RV, both univariate and multivariate logistic regression models demonstrated a significant joint effect that is greater than expected with an additive model. Moreover, RERI values were 3.765 and 4.365, respectively, which means that the joint effect of EV71 with norovirus and rotavirus was 3.765 and 4.365 times higher, respectively, than the sum of the single virus effects on the severe-HFMD patients. Each 58 % of AP% means that the joint effect on the severe patients of HFMD accounted for 58 % of the causative agents in the severe-HFMD patients.

To date, there are no effective vaccines or specific antiviral drugs available to control severe HFMD. Although multiple studies have emphasized the pathogenesis of severe HFMD through biological, molecular or epidemiological methods, this still needs to be further elucidated. EV71 has been recognized to be related to severe HFMD [15, 16]; however, in most cases, the virus cannot be detected in CSF or serum from patients with severe disease through virus isolation or RT-PCR, but can be detected in the stool [10], and the main reason for this may be that the viremia stage is difficult to detect. In addition, there is evidence that norovirus and rotavirus can continue to be shed in stools for weeks, and central nervous system involvement with these viruses have been observed in patients with or without associated convulsions, indicating that the viruses were potentially related to neurological complications [17–21]. In the present study, the joint effects of these viruses related to neurological complications were therefore reasonable to deduce, and co-circulation of EV71, rotavirus and norovirus may partly explain the pathogenesis of severe HFMD, although the pathogenic mechanism underlying this phenomenon remained to be discovered.

The following limitations of the present study should be considered: The intestinal tract is a complex environment, and the detection of viruses is not indicative of their pathogenic function in clinical neurologic complications. The genuine causative relationship needs to be confirmed by the positive detection or isolation of the virus in cerebrospinal fluid. Despite of this limitation, the detection of these viruses at least could act as a helpful marker for predicting a high risk of severe complications in FHMD cases, and this knowledge might enhance targeted treatment in clinical practice.

## **Contributorship statement**

Li-Juan Liu and Hong-Mei Xu wrote the manuscript and finished the lab work; Xiu-Jun Li, Jun Wang, Xian-Jun Wang and Shu-Jun Ding collected the specimens and sent them to the lab; Ying-Jie Zhang designed the manuscript. Fang Tang and Jing Wang gave advice when revising the manuscript.
